# *In Situ* Characterization of Splenic *Brucella melitensis* Reservoir Cells during the Chronic Phase of Infection in Susceptible Mice

**DOI:** 10.1371/journal.pone.0137835

**Published:** 2015-09-16

**Authors:** Delphine Hanot Mambres, Arnaud Machelart, Jean-Marie Vanderwinden, Carl De Trez, Bernhard Ryffel, Jean-Jacques Letesson, Eric Muraille

**Affiliations:** 1 Unité de Recherche en Biologie des Microorganismes, Laboratoire d’Immunologie et de Microbiologie, Université de Namur, Namur, Belgium; 2 Laboratory of Neurophysiology, Université Libre de Bruxelles, Campus Erasme, Brussels, Belgium; 3 Department of Molecular and Cellular Interactions, Vlaams Interuniversitair Instituut voor Biotechnologie, Vrije Universiteit Brussel, Gebouw E, Verdieping 8, Pleinlaan 2, 1050, Brussels, Belgium; 4 CNRS, UMR7355, Orleans, France; Experimental and Molecular Immunology and Neurogenetics, University of Orléans, Orléans, France; Institute of Infectious Disease and Molecular Medicine, IDM, Cape Town, South Africa; 5 Laboratoire de Parasitologie, Université Libre de Bruxelles, Campus Erasme, Bruxelles, Belgique; National University, COSTA RICA

## Abstract

*Brucella* are facultative intracellular Gram-negative coccobacilli that chronically infect humans as well as domestic and wild-type mammals, and cause brucellosis. Alternatively activated macrophages (M2a) induced by IL-4/IL-13 via STAT6 signaling pathways have been frequently described as a favorable niche for long-term persistence of intracellular pathogens. Based on the observation that M2a-like macrophages are induced in the spleen during the chronic phase of *B*. *abortus* infection in mice and are strongly infected *in vitro*, it has been suggested that M2a macrophages could be a potential *in vivo* niche for *Brucella*. In order to test this hypothesis, we used a model in which infected cells can be observed directly *in situ* and where the differentiation of M2a macrophages is favored by the absence of an IL-12-dependent Th1 response. We performed an *in situ* analysis by fluorescent microscopy of the phenotype of *B*. *melitensis* infected spleen cells from intranasally infected IL-12p40^-/-^ BALB/c mice and the impact of STAT6 deficiency on this phenotype. Most of the infected spleen cells contained high levels of lipids and expressed CD11c and CD205 dendritic cell markers and Arginase1, but were negative for the M2a markers Fizz1 or CD301. Furthermore, STAT6 deficiency had no effect on bacterial growth or the reservoir cell phenotype *in vivo*, leading us to conclude that, in our model, the infected cells were not Th2-induced M2a macrophages. This characterization of *B*. *melitensis* reservoir cells could provide a better understanding of *Brucella* persistence in the host and lead to the design of more efficient therapeutic strategies.

## Introduction

A growing number of clinically relevant infectious diseases have been reported to be characterized by pathogen persistence in the host. Chronic and recurrent infections implicate long-lasting and costly therapy and are the cause of significant morbidity in the world [1]. Strategies to cure persistent intracellular bacterial pathogens fail in part due to our poor understanding of the microenvironment necessary to sustain chronic infection. Brucellosis, considered one of the most common global and contagious zoonoses, affects a large range of mammals and is caused by facultative intracellular Gram-negative coccobacilli of the genus *Brucella* [2,3]. Human brucellosis is a severe and debilitating disease that may lead to permanent damage and requires prolonged and combined antibiotic therapy [4]. Direct cutaneous contact, ingestion of infected animal products and inhalation of airborne agents are the main routes of transmission in humans [5]. No safe or effective vaccine is available to prevent human infection [6].

Despite recent progress in mouse models of brucellosis, very little is known about the phenotype and the physiological state of cells chronically infected by *Brucella in situ*. Following intraperitoneal [7] or intranasal infection [8], the spleen appear to be stably and durably colonized by *Brucella*. However, in wild type mice, *Brucella* persist at levels which are too low in spleen (10^3^−10^5^ CFU at 28 days post-infection) for direct microscopic analysis of the phenotype of reservoir cells *in situ* (discussed in [9]) or to permit the purification of infected cells by flow cytometry. To bypass this problem, we have previously used highly susceptible IL-12p40^-/-^ BALB/c mice. In these mice, both of the protective Th1 and Th17 responses are severely impaired by the absence of functional IL-12 and IL-23 complexes. As a result, they display at least 10^6^ CFU/spleen during the chronic phase of infection. Using a *B*. *melitensis* 16M strain stably expressing the mCherry fluorescent protein (mCherry-Br), we have shown [9] that 12 days following intraperitoneal infection the reservoir cells are located in the T cell area of the white pulp and colocalize with CD11c, DEC-205 C-type lectin (CD205) (dendritic cells markers) and MOMA-1 (marginal zone macrophage marker) staining. A recent report from Xavier *et al*. [10] demonstrates that in wild type C57BL/6 mice the chronic phase of *B*. *abortus* infection is associated with the induction of alternatively activated (also called M2a) like macrophages and that these cells constitute a favorable niche *in vitro* for *Brucella* growth. Based on these data, Xavier *et al*. have suggested that “*B*. *abortus* survives and replicates preferentially in alternatively activated macrophages” during chronic infection in mice.

M2a polarization of macrophages is induced by the Th2 cytokines IL-4 and IL-13 through STAT6 dependent signaling (reviewed in [11]). M2a macrophages are characterized by the selective expression of various markers such as Arginase1 (Arg1), Fizz1 (Found in Inflammatory Zone 1) [12] and MGCL1 (Macrophage Galactose-C-type lectin, CD301) [13], and display low microbicidal activity and lipid oxidative metabolism (reviewed in [11,14–16]). Th2-induced M2a macrophages have been shown in various models of infection to constitute a favorable niche for long-term persistence of bacteria [17,18] and protozoa [19,20]. In order to test the Xavier *et al*. hypothesis [10]that M2a macrophages constitute the preferential reservoir cells for *Brucella in vivo* in the spleen, we chose to use highly susceptible IL-12p40^-/-^ BALB/c mice as a model in which *Brucella* infected cells can be observed directly *in situ* by fluorescence microscopy and where M2a macrophage differentiation is favored by the absence of an IL-12-dependent Th1 response. In this experimental model, we analyzed *in situ* the phenotype of *Brucella melitensis* infected cells in spleens and the impact of STAT6 deficiency, known to block macrophage polarization to the M2a phenotype [21–23], on this phenotype. We observed that the absence of the IL-4/IL-13 signaling pathways in IL-12p40^-/-^ STAT6^-/-^ mice does not affect the course of *Brucella* infection or the phenotype of reservoir cells when compared to IL-12p40^-/-^ mice.

## Materials and Methods

### Ethics Statement

The procedures in this study and the mice handling complied with current European legislation (directive 86/609/EEC) and the corresponding Belgian law “Arrêté royal relatif à la protection des animaux d'expérience du 6 avril 2010 publié le 14 mai 2010". The Animal Welfare Committee of the Université de Namur (UNamur, Belgium) has reviewed and approved the complete protocol (Permit Number: 05–558).

### Mice and reagents

Wild-type BALB/c mice were acquired from Harlan (Bicester, UK). We used also STAT6^-/-^ BALB/c mice (strain C.129S2-*STAT6* ^*tm1Gru*^/J), IL12p40^-/-^ BALB/c mice (C.129S1-*IL12b* ^*tm1Jm*^/J), both purchased from Jackson Laboratory, and the double knockout STAT6/IL12p40 BALB/c mice, obtained by a cross between the two single-gene knockout strains cited above. All wild-type and deficient mice used in this study were bred in the animal facility of the Gosselies campus of the Université Libre de Bruxelles (ULB, Belgium).

We used a strain of *Brucella melitensis* 16M stably expressing a rapidly maturing variant of the red fluorescent protein DsRed [24]: the mCherry protein (mCherry-Br), under the control of the strong *Brucella* spp. promoter, PsojA. Construction of the mCherry-Br strain has been described previously in detail [9]. It is grown in biosafety level III laboratory facilities. The cultures were grown overnight with shaking at 37°C in 2YT media (Luria-Bertani broth with double quantity of yeast extract) and were washed twice in RPMI 1640 (Gibco Laboratories) (3500 x g, 10 min.) before inoculation in the mice.

### Mice infection

Mice were anaesthetized with a cocktail of Xylazine (9 mg/kg) and Ketamine (36 mg/kg) in PBS before being inoculated intranasally (i.n.) with 2x10^7^ CFU of mCherry-expressing *B*. *melitensis* in 30 μL of RPMI. Control animals were inoculated with the same volume of RPMI. The infectious doses were validated by plating serial dilutions of inoculums. At the selected time after infection, the mice were sacrificed by cervical dislocation. Immediately after sacrifice, the spleen, liver and lung cells were collected for bacterial count, flow cytometry and/or microscopic analyses.

### Bacterial count

Spleens, livers and lungs were crushed and transferred to PBS/0.1% X-100 triton (Sigma). We performed successive serial dilutions in RPMI to get the most accurate bacterial count and plated them on 2YT medium. The CFU were counted after 5 days of culture at 37°C.

### Immunofluorescence microscopy

Spleens were fixed for 4 hours at room temperature in 2% paraformaldehyde (pH 7.4), washed in PBS, and incubated overnight at 4°C in a 20% PBS-sucrose solution. The tissues were then embedded in Tissue-Tek OCT compound (Sakura), frozen in liquid nitrogen, and cryostat sections (thickness, 5 μm) were prepared. For the staining, tissue sections were rehydrated in PBS and incubated in a PBS solution containing 1% blocking reagent (Boeringer) (PBS-BR 1%) for 20 minutes before being incubated overnight in PBS-BR 1% containing mAbs or reagents: DAPI nucleic acid stain Alexa Fluor 350 or 488 phalloidin (Molecular Probes) to visualize the structure of the organ, and Allophycocyanin (APC)-coupled BM8 (anti-F4/80, Abcam), Alexa Fluor 647-coupled M1/70 (anti-CD11b, BD Biosciences), Alexa Fluor 647-coupled HL3 (anti-CD11c, BD Biosciences), Alexa Fluor 647-coupled 53–6.7 (anti-CD8α, Santa Cruz Biotechnology), Alexa Fluor 647-coupled RB6-8C5 (anti-Gr1, eBioscience), APC-coupled M5/114.15.2 (anti-MHCII, I-A/I-E), Alexa Fluor 647-coupled NLDC-145 (anti-Dec205/CD205, BioLegend), Alexa Fluor 647-coupled ER-MP23 (anti-CD301, AbD Serotec), Bodipy 493/503 (Molecular Probes), Biotin-coupled HL3 (anti-CD11c, BD Biosciences), Biotin-coupled RB6-8C5 (anti-Gr1, eBioscience), Biotin-coupled MOMA1 (anti-Marginal Zone Macrophages, BMA Biomedicals), Biotin-coupled ERTR9 (anti-SIGN-R1, BMA Biomedicals), IgG H-52 (anti-Arg1, Santa Cruz Biotechnology), and IgG Fizz (anti-RELMα, Abcam) to stain the cells of interest. Incubation with a streptavidin-coupled fluorochrome for 2 hours was necessary for the biotin-coupled Ab: Alexa Fluor 350 or Alexa Fluor 647 streptavidin (Molecular Probes). Incubation with a secondary antibody Alexa Fluor 647-coupled goat anti-rabbit IgG (Molecular Probes) was necessary for the anti-Arg1 and anti-Fizz mAbs. Slides were mounted in Fluoro-Gel medium (Electron Microscopy Sciences, Hatfield, PA). Labeled tissue sections were visualized with an Axiovert M200 inverted microscope (Zeiss, Iena, Germany) equipped with a high-resolution monochrome camera (AxioCam HR, Zeiss). Images (1384x1036 pixels, 0.16μm/pixel) were acquired sequentially for each fluorochrome with A-Plan 10x/0.25 N.A. and LD-Plan-NeoFluar 63x/0.75 N.A. dry objectives and recorded as eight-bit grey-level *.zvi files. At least 3 slides per organ were analyzed from 3 different animals and the results are representative of 2 independent experiments.

### Confocal microscopy

Confocal analyses were performed using the LSM780 confocal system fitted on an Observer Z1 inverted microscope equipped with an alpha Plan Apochromat 63x/1.46 NA oil immersion objective (Zeiss, Iena, Germany). Hoechst/DAPI was excited using a 405 nm blue diode, and emission detected using a band-pass filter (410–480 nm). For detection of the green fluorochrome GFP, the 488 nm excitation wavelength of the Argon/2 laser was used in combination with a band-pass emission filter (BP500-535 nm). For the red fluorochrome mCherry, the 543nm excitation wavelength of the HeNe1 laser, and a band-pass emission filter (BP580-640 nm) were used. For far-red fluorochromes such as APC and Alexa Fluor® 647, the 633 excitation wavelength of the HeNe2 laser, and a band-pass emission filter (BP660-695 nm) were used. To ensure optimal separation of the fluorochromes, blue & red signals were acquired simultaneously in one track and green & far-red signals in a second track. The electronic zoom factor and stack depth were adjusted to the region of interest while keeping image scaling constant (x-y: 0.066 micron, z: 0.287 micron). A line average of 8 was used and datasets were stored as 8-bit proprietary *.czi files. The images were displayed using Zen2012 software (Zeiss) with linear manual contrast adjustment and exported as 8-bit uncompressed *.TIF images. The figures were prepared with the Canvas program.

### PCR verification of IL-12p40, STAT-6 and IL-12p40/STAT-6 deficient mice

DNA was extracted from the tail of the mouse using the High Pure PCR Template Preparation Kit (ROCHE). The extracted DNA was mixed with Green buffer 5x, dntp 5mM, MgCl_2_ 25mM, H_2_O, GoTaq polymerase (2.5mM each) and primers. Primers used to amplify the STAT-6 gene (5'-3'): AGTGGGTCCCCTTCACTCT (IMR7416 wt), CTCCGGAAAGCCTCATCTT (IMR1822 common), ATCCATCTTGTTCAATGGCCGATC (IMR0092 Neo). Primers used to amplify the IL-12p40 gene (5'-3'): AGTGAACCTCACCTGTGACACG (IMR0457 wt), TCTTTGCACCAGCCATGAGC (IMR0458 common), CTTGGGTGGAGAGGCTATTC (IMR6916 Neo). The PCR cycling parameters were as follows: denaturation at 94°C for 3 min, annealing at 66°C for 1 min and extension at 72°C for 1 min. 35 cycles were programmed. The PCR products were checked on 1% agarose gels in 1x TAE buffer.

### Statistical analysis

We used a (Wilcoxon-) Mann-Whitney test provided by the GraphPad Prism software to statistically analyze our results. Each group of deficient mice was compared to wild-type mice. We also compared each group with each other and displayed the results when required. Values of p < 0.05 were considered to represent a significant difference. *, **, *** denote p<0.05, p<0.01, p<0.001, respectively.

## Results

### STAT6 deficiency does not affect the control of *Brucella* infection in IL-12p40^-/-^ mice

**Fig 1:**
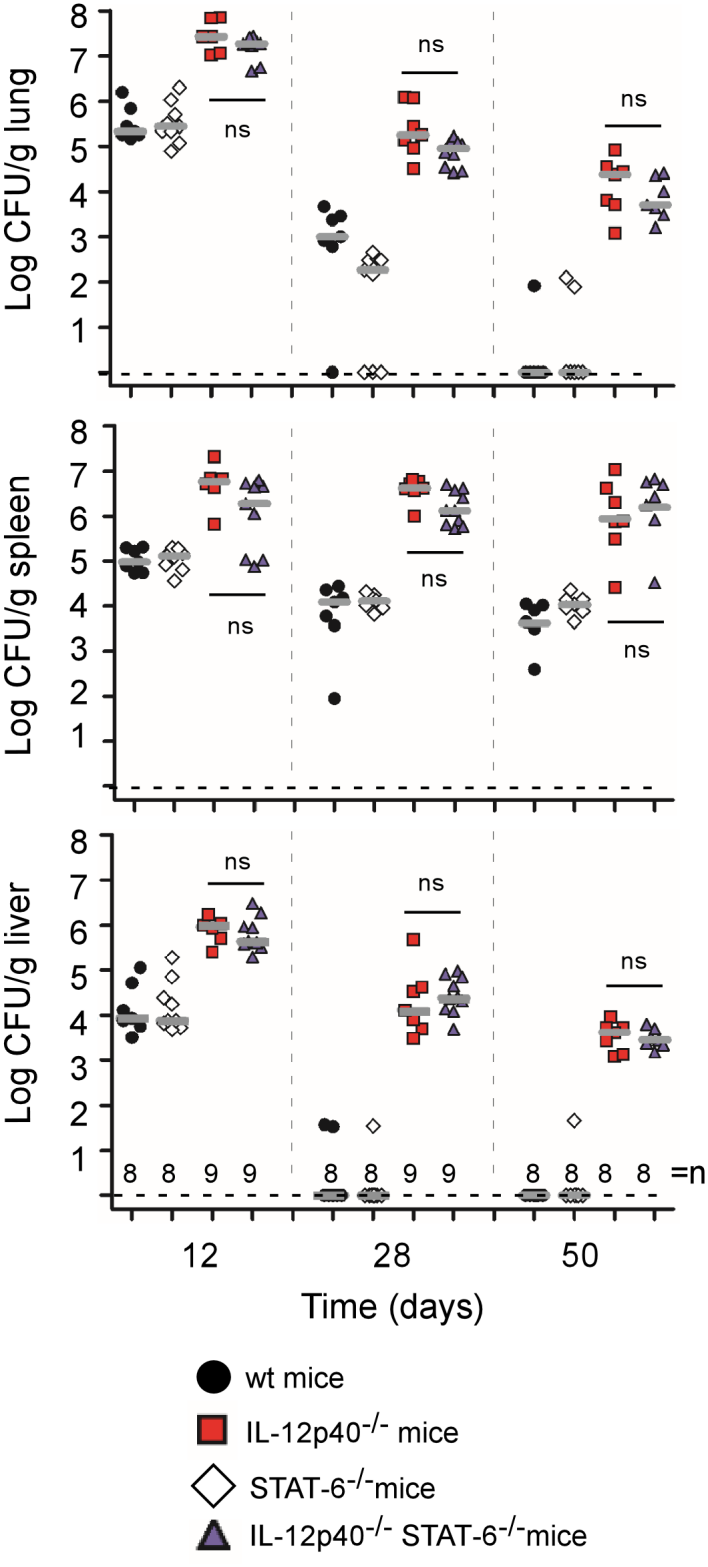
Course of *B*. *melitensis* infection in organs of wild-type (wt), STAT6-, IL12p40- and STAT6/IL12p40-deficient BALB/c mice. The mice were injected intranasally (i.n.) with 2x10^7^ CFU of mCherry-Br *B*. *melitensis* and sacrificed at the indicated times. The data represent the number of CFU per gram of lung, spleen and liver. Grey bars represent the medians. “n” is the number of mice used. These results are representative of at least two independent experiments. ns, non-significant.

STAT6 is required to mediate the response to IL-4 [23] and IL-13 [22] and develop the Th2 response [21]. It is well established that its deficiency completely abolishes IL-4/IL-13 induced M2a macrophage polarization *in vivo* [21–23]. We compared IL-12p40^-/-^ BALB/c mice with IL-12p40^-/-^ STAT6^-/-^ BALB/c mice in a model of intranasal infection by 2x10^7^ CFU of mCherry-Br. Wild-type and STAT6^-/-^ BALB/c mice were used as internal controls. The absence of functional STAT6 and IL-12p40 genes in each group of deficient mice was confirmed by PCR (S1 Fig). The CFU counts in the lung, spleen and liver were analyzed at 12, 28 and 50 days post-infection (Fig 1). Confirming previous results in the intraperitoneal infectious model [7], wild-type and STAT6^-/-^ mice displayed similar CFU counts, whatever the organ or the time tested. We observed that the absence of a functional STAT6 signaling pathway, which impairs the development of a Th2 response, does not affect the control of infection even in absence of a Th1 response in IL-12p40. Similar results were obtained with a dose of 2x10^4^ CFU (data not shown).

### STAT6 deficiency does not modify the phenotype of *Brucella* infected cells in IL-12p40^-/-^ mice

A minimum of 10^6^ CFU/g of organ is necessary to observe mCherry-Br *in situ* by fluorescent microscopy [9]. In our model, this level is reached only in the spleen during the chronic phase of infection, not in the lung or liver. For this reason, we limited our characterization of *Brucella* infected cells to this organ.

**Fig 2:**
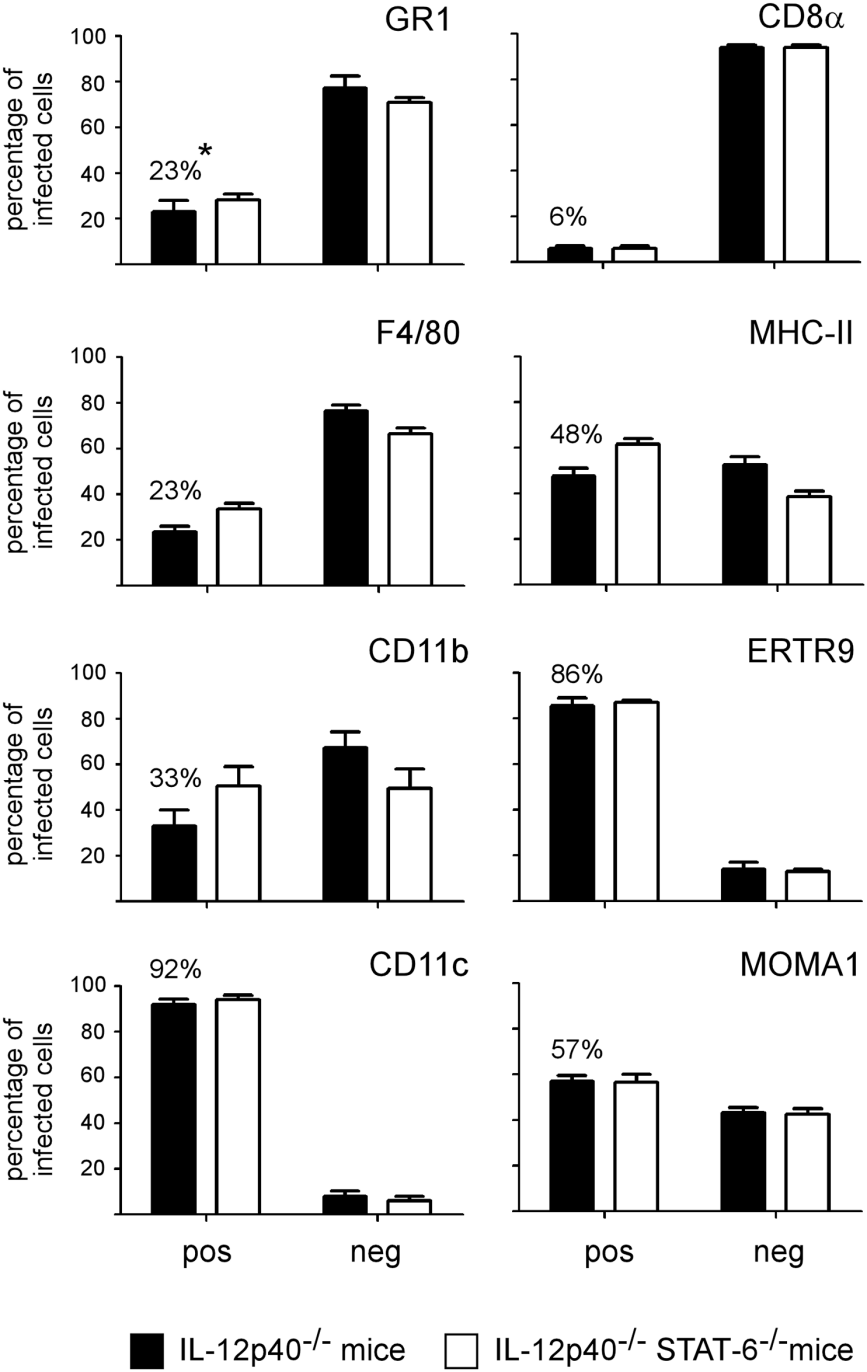
Comparison of the phenotype of infected cells in the spleen of IL12p40- and IL12p40/STAT6- deficient BALB/c mice. IL12p40- and IL12p40/STAT6-deficient BALB/c mice were injected i.n. with 2x10^7^ CFU of mCherry-Br. The mice were sacrificed at 28 days post-infection and the spleens were collected and examined by immunohistofluorescence. The data represent a comparative analysis of the percentage of mCherry-Br that co-localize or not with GR1-, F4/80-, CD11b-, CD11c-, CD8α-, MHCII-, ER-TR9- and MOMA-1- expressing cells in the two lineages of mice. These results are representative of at least three independent experiments. *: percentage of co-localization between mCherry-Br and positive cells for the antigen in IL12p40^-/-^ STAT6^+/+^ mice.

**Fig 3:**
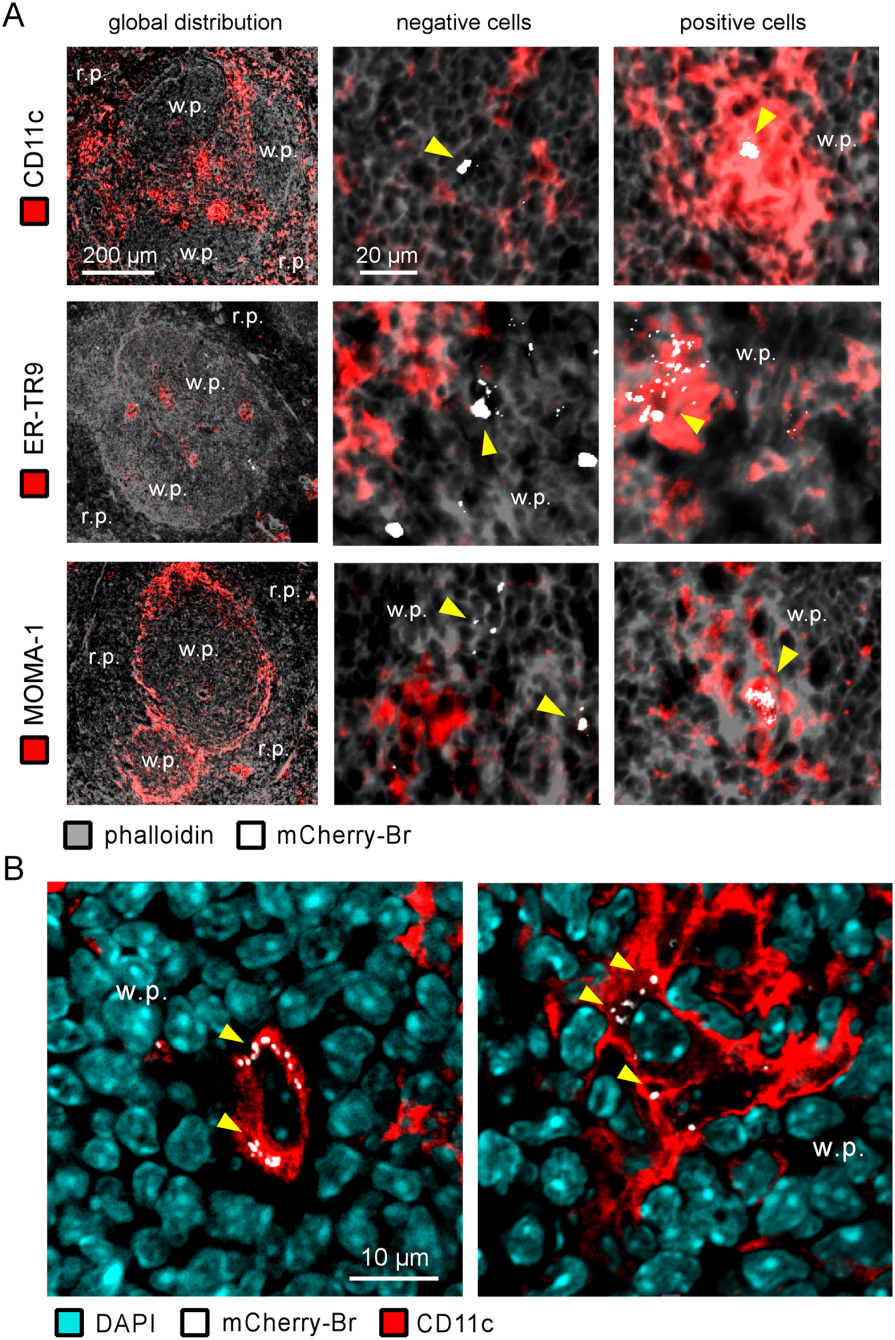
Characterization of the cell surface phenotype of infected cells in the spleen of IL12p40-deficient BALB/c mice. IL12p40^-/-^ STAT6^+/+^ BALB/c mice were injected i.n. with 2x10^7^ CFU of mCherry-Br. The mice were sacrificed at 28 days post-infection and the spleens were collected and examined by immunohistofluorescence. **A**. The left panels show the overall distribution of the CD11c-, ERTR9 and MOMA-1-expressing cells in the spleen. The panels to the right of the first ones show mCherry-Br co-localization with negative cells and positive cells for CD11c, ER-TR9 and MOMA-1. The panels are color-coded with the text for phalloidin, the antigen examined or mCherry-Br. Scale bar = 200 and 20 μm, as indicated. r.p.: red pulp; w.p.: white pulp. Yellow arrowheads indicate the presence of bacteria. The data are representative of at least three independent experiments. **B**. Representative confocal images of mCherry-Br infected CD11c^+^ cells. The panels are color-coded with the text for DAPI, mCherry-Br or CD11c. Scale bar = 10 μm, as indicated. w.p.: white pulp.

In order to determine the impact of STAT6 deficiency on the phenotype of infected spleen cells, we compared *in situ* using fluorescence microscopy the expression of various cell surface markers on infected cells in the spleen from IL-12p40^-/-^ and IL-12p40^-/-^ STAT6^-/-^ BALB/c mice at 28 days post-infection. We analyzed the expression of GR1, F4/80, CD11b, CD11c, CD8α, MHC-II, MOMA-1 and ER-TR9 markers on a minimum of 200 infected cells from 3 animals per group. This analysis was performed under blinded conditions to reduce the risk of biased results, and it was repeated in two distinct experimental infections. The frequency of each negative and positive infected cells from both mouse strains is presented in Fig 2. No significant difference was observed between the IL-12p40^-/-^ STAT6^+/+^ and IL-12p40^-/-^ STAT6^-/-^ mice, whatever the markers tested. In consequence, we only present images from IL-12p40^-/-^ STAT6^+/+^ mice to illustrate each staining. The general distribution for each staining and an example of negative and positive cells is presented in Fig 3A and S2 Fig. In order to further confirm the expression of CD11c, MOMA-1 and ER-TR9 by infected cells, a confocal analysis was also performed on the spleen section (Fig 3B for CD11c, data not shown for MOMA-1 and ER-TR9).

As reported previously following intraperitoneal infection [9], infected cells were mainly found during the chronic phase of infection in the white pulp of the spleen and preferentially in the T cell area (data not shown).

Recruitment of GR1^+^ cells (presumably neutrophils, based on 2A8 (Ly-6G specific) co-staining (data not shown) and characteristic nuclei visualized by DAPI staining) in the red and white pulp was observed in infected mice compared to naive mice. However, as previously observed [9], only rare infected cells co-localized with GR1 staining. Most of them expressed low GR1 staining and were observed in dense aggregates of cells, which made it difficult to precisely identify the infected cells.

CD11c, a marker mainly expressed by dendritic cells (DC) in the spleen [25], was expressed at high or low levels on 92% of infected cells. The low frequency of infected cells expressing CD11b and F4/80 (generally at low level) suggests that these CD11c^+^ cells do not belong to the "inflammatory DC" subset, known to co-express CD11b, CD11c and F4/80 markers at high levels *in situ* [26,27], or the "myeloid related" CD8α^−^ CD11b^+^ classical DC subset (reviewed in [28]). As expected, CD11b staining strongly co-localized with GR1 and 2A8 staining (data not shown), suggesting that a majority of the infected CD11b^+^ cells were neutrophils.

In the spleen of naive mice, CD8α was mainly expressed on T cells and a subset of DC (termed "lymphoid-related" CD8α^+^ CD11b^−^ DC" [28]) and localized mainly in the T cell area of the white pulp. In our model, very rare infected cells co-localized with CD8α staining (6%) and the majority expressed only low levels of CD8α, suggesting that infected CD11c^+^ cells do not belong to the lymphoid-related DC. Interestingly, MHCII, generally expressed at high levels on splenic DC [29], was mainly expressed at low levels on infected cells. Numerous infected CD11c^+^ cells expressed low or undetectable levels of MHCII (S3 Fig), suggesting that *Brucella* infection could reduce the expression of MHC-II on DC *in vivo* as recently described for alveolar macrophage *in vitro* [30].

A strong association between ER-TR9 (SIGN-R1), MOMA-1 markers and infected cells was also observed. In naive mice, these markers are mainly present in the marginal zone of spleen and identify marginal zone macrophages (MZM) and metallophilic marginal zone macrophages (MMZM), respectively [31]. Surprisingly, co-localization between CD11c and low levels of MOMA-1 (A in S4 Fig) and ER-TR9 (data not shown) on infected cells was observed frequently in the spleen of infected mice. This phenomenon was mainly found in dense aggregate of cells in the white pulp.

### *Brucella* infected cells do not express typical alternatively activated markers such as Fizz1 and CD301

**Fig 4:**
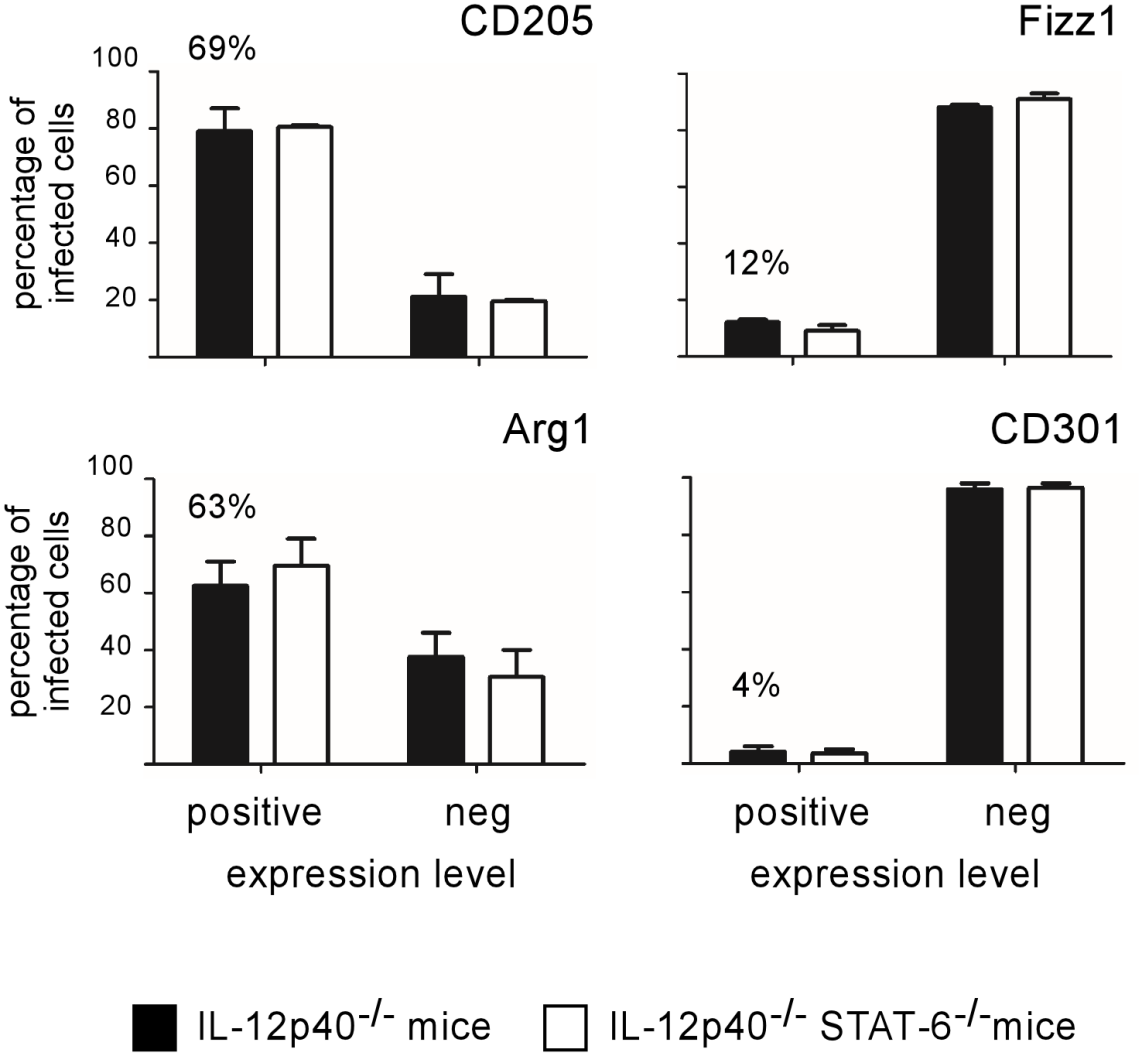
Analysis of CD205, Arginase1, Fizz1 and CD301 expression on infected spleen cells from IL12p40- and STAT6/IL12p40-deficient BALB/c mice. IL12p40^-/-^ STAT-6^+/+^ and IL12p40^-/-^ STAT-6^-/-^ BALB/c mice were injected i.n. with 2x10^7^ CFU of mCherry-Br. The mice were sacrificed at 28 days post-infection and the spleens were collected. The figure shown the percentage of mCherry-Br that co-localizes or not with Dec205-, Arg1-, Fizz1- and CD301-expressing cells in the spleen of IL12p40^-/-^ STAT-6^+/+^ and IL12p40^-/-^ STAT-6^-/-^ BALB/c mice. The percentage of co-localization between mCherry-Br and positive cells for the antigen in IL12p40^-/-^ STAT-6^+/+^ mice is indicated. The data are representative of at least two independent experiments.

**Fig 5:**
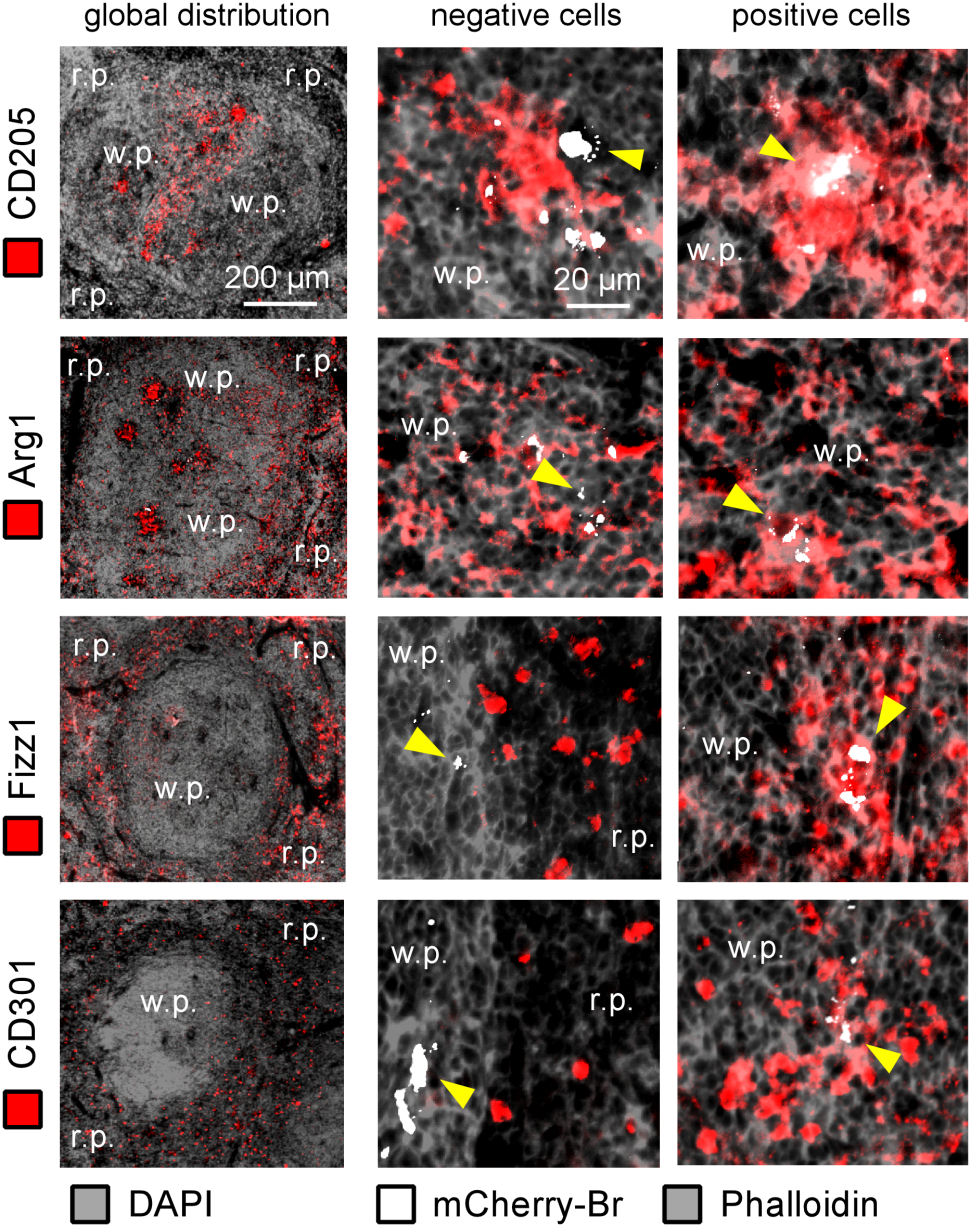
CD205, Arginase1, Fizz1 and CD301 expression on infected spleen cells from IL12p40-deficient BALB/c mice. IL12p40^-/-^ STAT6^+/+^ BALB/c mice were injected i.n. with 2x10^7^ CFU of mCherry-Br. The mice were sacrificed at 28 days post-infection and the spleens were collected and examined by immunohistofluorescence. for the expression of CD205 and M2 markers and co-localization with mCherry-Br. The left panels show the overall distribution of the Dec205-, Arg1-, Fizz1- and CD301-expressing cells in the spleen. The middle panels show mCherry-Br co-localization with negative cells for Dec205, Arg1, Fizz1 and CD301. The right panels present the immunofluorescence analysis of mCherry-Br co-localization with positive cells for Dec205, Arg1, Fizz1 and CD301. The panels are color-coded with the text for DAPI, phalloidin, the antigen examined or mCherry-Br. Scale bar = 200 and 20 μm, as indicated. r.p.: red pulp; w.p.: white pulp. Yellow arrowheads indicate the presence of bacteria. The data are representative of at least two independent experiments.

In order to more thoroughly characterize the phenotype of infected cells, we analyzed the expression of the DC marker DEC-205 (CD205) [32] and of several well-established M2 differentiation markers such as Arginase1 (Arg1), Fizz1 and CD301 (reviewed in [11]) (Fig 4). Infected cells frequently co-localize with CD205 (69%) and Arg1 (63%) staining. The majority of highly infected cells (displaying large aggregates of mCherry-Br) expressed high levels of both markers (Fig 5). The frequent expression of CD205 and Arg1 markers by infected cells has been confirmed by confocal analysis (data not shown). In contrast, Fizz1 and CD301 staining displayed weak co-localization with mCherry-Br signal staining (Fig 4) and was mainly located in the red pulp and the marginal zone (Fig 5). Fizz1^+^ or CD301^+^ infected cells are observed very rarely in the white pulp. As previously observed for other markers, no significant difference was observed between STAT6^+/+^ and STAT6^-/-^ mice for all markers tested (Fig 4).

**Fig 6:**
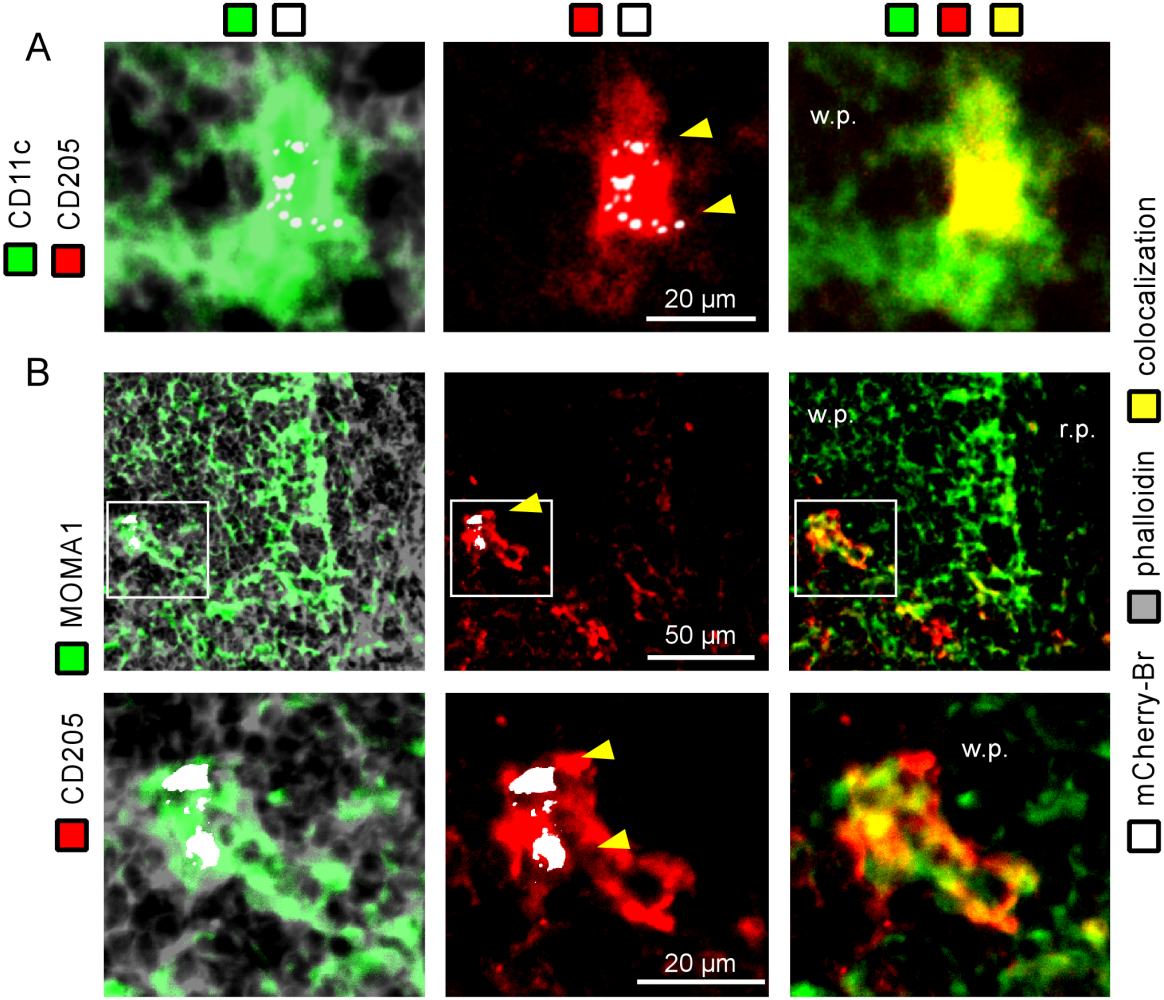
Characterization of infected cells expressing CD11c, CD205 and MOMA-1 in the spleen of IL12p40-deficient BALB/c mice. IL12p40^-/-^ STAT6^+/+^ BALB/c mice were injected i.n. with 2x10^7^ CFU of mCherry-Br. The mice were sacrificed at 28 days post-infection and the spleens were collected and examined by immunohistofluorescence. **A**, The left panel shows mCherry-Br co-localization with cells expressing CD11c. The middle panel shows mCherry-Br co-localization with cells expressing CD205 and the right panel shows co-localization of CD11c- and CD205-expressing cells. **B**, The upper panels show distribution of MOMA-1-expressing cells and co-localization with mCherry-Br (left), distribution of DEC205-expressing cells and co-localization with mCherry-Br (middle), and co-localization of MOMA-1- and CD205-expressing cells (right). The panels below are higher magnification views of the same stainings. The panels are color-coded with the text for phalloidin, the antigen examined or mCherry-Br. Scale bar = 50 and 20 μm, as indicated. r.p.: red pulp; w.p.: white pulp. Yellow arrowheads indicate the presence of bacteria. The data are representative of at least two independent experiments.

As expected, strong co-localization of CD11c and CD205 on infected cells was observed (Fig 6A). Similarly, staining for MOMA-1 (Fig 6B) and ER-TR9 (data not shown) co-localized with CD205 staining on infected cells. It was interesting to see that CD205 expression on MOMA-1 cells was mainly found around infected cells and not on MOMA-1^+^ non-infected cells located in the marginal zone (Fig 6B). Similar results were observed for ER-TR9 expression (data not shown).

### *Brucella* infected cells are lipid rich

**Fig 7:**
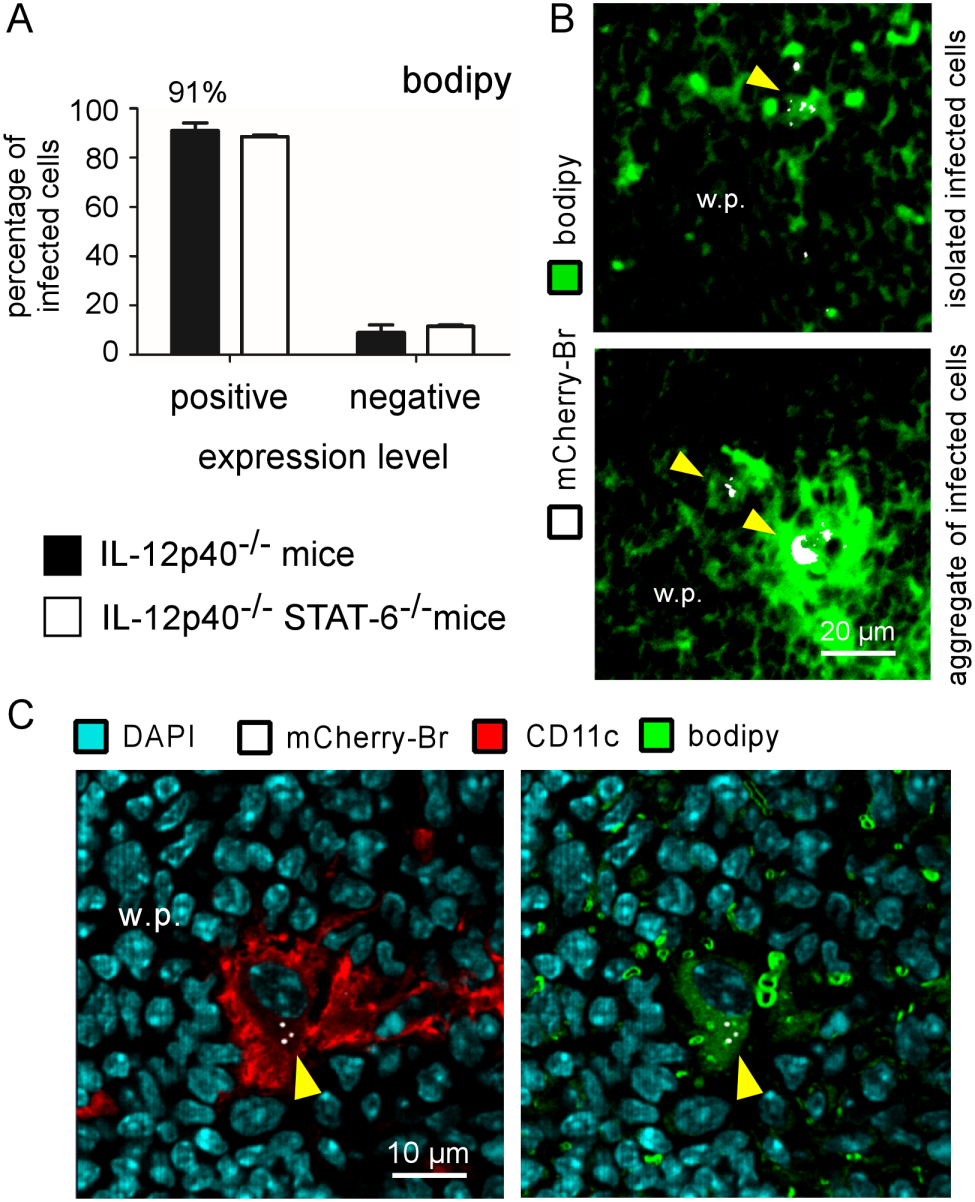
Characterization of the lipid storage of infected cells in the spleen of IL12p40-deficient and STAT6/IL12p40-deficient BALB/c mice. IL12p40^-/-^ STAT6^+/+^ and IL12p40^-/-^ STAT6^-/-^ BALB/c mice were injected i.n. with 2x10^7^ CFU of mCherry-Br. The mice were sacrificed at 28 days post-infection and the spleens were collected and examined by immunohistofluorescence. **A**, The data represent a comparative analysis of the percentage of mCherry-Br that co-localizes or not with cells stained with Bodipy. The percentage of co-localization between mCherry-Br and cells stained with Bodipy in IL12p40^-/-^ STAT-6^+/+^ mice is indicated. **B**, Co-localization of mCherry-Br and cells stained with Bodipy in IL12p40^-/-^ STAT-6^+/+^ mice **C**, Representative confocal images of mCherry-Br infected CD11c^+^ cells stained with Bodipy in IL12p40^-/-^ STAT-6^+/+^ mice. The left picture shows co-localization between CD11c staining and mCherry-Br, the right picture shows co-localization between Bodipy staining and mCherry-Br. The panels are color-coded with the text for DAPI, the antigen examined, Bodipy or mCherry-Br. Scale bar = 20, and 10 μm, as indicated. r.p.: red pulp; w.p.: white pulp. Yellow arrowheads indicate the presence of bacteria. The data are representative of at least two independent experiments.

In order to learn more about the metabolism of *Brucella* infected cells, we used the lipophilic Bodipy dye [33,34] to analyze fat storage alteration in infected cells (Fig 6). Very strong co-localization between infected cells and Bodipy staining was observed in both strains of mice (Fig 7A). This co-localization was observed on both isolated cells and cells within aggregates (Fig 7B). Confocal analysis confirmed that Bodipy staining co-localized with CD11c staining on infected cells (Fig 7C). Note that Bodipy does not seems to stain specifically the bacteria. As expected, microscopic analysis showed that Arg1 staining also co-localized with Bodipy staining (B in S4 Fig) on infected cells.

## Discussion

Recent evidence suggests that persisting intracellular pathogens are able to reprogram the host cellular microenvironment to their advantage. They frequently polarize the phenotype of host cells to produce an anti-inflammatory and nutrient-rich environment assuring their long term persistence in the host (reviewed in [11,14–17]). In this new paradigm, it will be crucial to identify and characterize the phenotype of the main reservoir cells for bacteria during chronic infection and to define the signaling pathways implicated in their polarization during infectious processes.

Bacteria of the genus *Brucella* have been long studied as a relevant experimental model of chronic infections in animals and humans due to their great impact on both husbandry practice and human health worldwide [35]. The mouse is considered as a useful animal model to investigate the pathogenesis of brucellosis, to identify specific virulence factors of *Brucella* spp. and to characterize the host immune response [35]. However, direct observation of *Brucella* infected cells in tissues of immunocompetent mice is a formidable technical challenge due to the very low levels of *Brucella* persistence during the chronic phase of infection. To date, the only studies directly analyzing the phenotype of *Brucella* infected cells *in vivo* in wild-type mice have been performed on mice infected for less than 5 days [9,36]. Wild-type BALB/c and C57B/6 mice present approximately 10^3^−10^4^ CFU per spleen at 28 days post-infection [7] suggesting that at most 5x10^3^ cells are infected per 5x10^7^ spleen cells (1/10,000). Furthermore, this stills probably an overestimation because it seems unlikely that reservoir cells would only contain one bacterial cell. Due to this very low frequency, infected cells are not easily observed directly by microscopy. To bypass this problem, we previously used highly susceptible IL-12p40^-/-^ BALB/c mice that display at least 10^6^ CFU in the spleen from 12 to 28 days post-intraperitoneal infection [9]. In the present study, IL-12p40^-/-^ BALB/c mice were infected intranasally and the phenotype of the infected spleen cells was analyzed by fluorescent microscopy at 28 days post-infection. Our analysis confirms previous observations in the intraperitoneal infection model [9], showing that infected cells mainly localize in the white pulp and frequently express CD11c (92%) and CD205 (69%) receptors, two well-defined markers of the DC population. The absence of CD11b and F4/80 staining demonstrates that these cells are mostly distinct from classical myeloid-related CD8α^−^ CD11b^+^ DC present in the spleen of naive mice [28] and CD11b^+^ inflammatory DC observed during the acute phase of infection in resistant mice [9]. The absence of CD8α expression showed that they are also distinct from the CD8α^+^ DEC205^+^ lymphoid-related DC subset located in the T cell area of naive mice [28]. Expression of MOMA-1 or SIGN-R1/ER-TR9 on a fraction of CD11c^+^ infected cells suggests that part of these cells could derive from the marginal zone and differentiate from MOMA-1^+^ MMM and ER-TR9^+^ MZM. Like DC, both MMM and MZM are known to migrate from the marginal zone to the T cell area in response to infection by *Listeria monocytogenes* [31].

Unfortunately, in our infectious model, the microscopic analysis could not be completed with a flow cytometry analysis of the infected spleen cells. The classical 488-nm (blue) laser provides only minimal excitation (8%) at the very edge of the excitation spectrum of mCherry expression. In our bacterial strain, mCherry is expressed from a single chromosomal copy in order to conserve the full virulence of the bacteria [9] and allow for its long-term persistence in mice. This level of expression was not sufficient to distinguish the infected cells by flow cytometry among myeloid spleen cells displaying non-negligible auto-fluorescence (data not shown). The use of *Brucella*-specific antibodies to purify or analyze infected cells *ex vivo* is not a reliable alternative, as antibodies are not able to distinguish between infected cells and cell cross-presenting *Brucella* antigens. In a previous study [9], we observed that *Brucella*-specific antibodies detected both mCherry-Br^+^ cells and an important number of mCherry-Br^−^ cells in the spleen of infected mice. In addition, *ex vivo* observations following cell purification by flow cytometry are frequently a source of artifactual results. The frequency and phenotype of infected cells are inevitably altered by mechanical stress due to tissue destruction and centrifugation, as well as by exposition to collagenases and the cell incubation periods. To avoid these problems in our study, despite its evident limitations, we have privileged direct microscopic observation of infected cells *in situ*.

Taken together, the data from our microscopic analysis show that *Brucella melitensis* reservoir cells during the chronic phase of infection in highly susceptible IL-12p40^-/-^ BALB/c mice are all of myeloid origin. However, these reservoir cells express a phenotype distinct from classical DC and macrophages described in the spleen of naive mice, suggesting that *Brucella* infection directly or indirectly drives the differentiation of these cells. The phenotype of the main type of *Brucella* infected spleen cells (92% CD11c^+^, 69% CD205^+^, 91% Bodipy^+^, 63% Arg1^+^) is strongly reminiscent of the "foamy cell" phenotype observed in the lung during *Mycobacterium tuberculosis* infection. These cells display a heterogeneous phenotype depending on the host species, but are found to express high levels of both CD11c and CD205 markers, low levels of MHC-II [37], and display high levels of Arg1 [38] and lipid bodies [39].

Recent work from Xavier *et al*. [10] has shown that M2a macrophages (identified by CD301 and Fizz1 expression *in situ*) are induced during the chronic phase of *Brucella abortus* infection in wild-type C57BL/6 mice. Based on the observation that M2a macrophages are strongly infected *in vitro* and that the PPAR agonist, known to induce M2a macrophage polarization [40], can affect the course of infection, Xavier *et al*. [10] have concluded that “*B*. *abortus* survives and replicates preferentially in alternatively activated macrophages” during chronic infection in mice. In the majority of infectious and non-infectious experimental models [21–23], Th2-induced M2a polarization of macrophages appears to be dependent on the STAT6 signaling axis. In our model, we observed that intranasal *B*. *melitensis* infection of IL-12p40^-/-^ BALB/c mice induces recruitment of M2a-like macrophages in the red pulp (identified *in situ* by the expression of both typical Fizz1 and CD301 M2a markers), but that *B*. *melitensis* infected cells localize mainly in the white pulp and do not express these markers. In addition, the course of infection and the phenotype of the infected cells do not appear to be affected by STAT6 deficiency. Taken together, these results demonstrate that, even though *B*. *melitensis* infected cells express high levels of Arg1, they cannot be formally considered as "classical" M2a macrophages dependent on the Th2 response and STAT6 signaling pathways. These results do not invalidate the work of Xavier *et al*. [10] as the bacterial strain, the mouse strain and the infection protocol are not comparable.

*Mycobacterium bovis* bacillus Calmette-Guérin (BCG) has been shown to induce Arg1 expression in STAT-6^-/-^ macrophages via alternative TLR/MyD88-dependent pathways [41] and Mycobacteria-infected macrophages produce soluble inflammatory factors, including IL-6, IL-10, and granulocyte colony-stimulating factor (G-CSF), that induce the expression of Arg1 in an autocrine/paracrine manner [42]. In addition, lactic acid produced by tumor cells has been recently demonstrated to induce Arg1 expression in macrophages via an hypoxia-inducible factor 1α (HIF1α)-dependent pathway [43] and that IL-6 produced by fibroblasts in a model of pulmonary hypertension induce Arg1 in macrophages via STAT3- and C/EBPβ-dependent pathways [44]. Thus, the production of Arg1 in macrophages appears to be controlled by several STAT6-independent pathways, which leads us to conclude that Arg1 expression alone can no longer be considered as a specific marker of M2a macrophages.

In a previous study [45], we demonstrated that *B*. *melitensis* activates the TLR9/MyD88 signaling pathways to induce NO production by infected inflammatory dendritic cells. NO is toxic to both the host tissues and invading pathogens, and its regulation is key to the suppression of host cytotoxicity. Induction of Arg1 expression via a TLR/MyD88-dependent pathway in response to the detection of bacteria could constitute a feedback mechanism that limits the toxicity of the immune response during chronic infection. However, this mechanism could also promote intracellular bacteria persistence by restricting NO production and constitute an escape immune mechanism for intracellular pathogens. In keeping with this hypothesis, Arg1-deficient mice had lower *M*. *tuberculosis* counts in the lung compared to wild-type mice [41] and *Brucella* growth is favored *in vivo* in the absence of NO production [45]. In addition to decreasing NO production by reducing L-Arginine availability, Arg1 produces L-Ornithine, the precursor of polyamines, such as putrescine, spermidine and spermine (reviewed in [46]). These latter have been described as anti-inflammatory agents in numerous models [47,48] and could be required for *Brucella* growth *in vivo* (discussed in [49]). Thus, expression of Arg1 in reservoir cells could lead to an immunosuppressive microenvironment that favors the persistence of intracellular bacteria such as *Brucella*.

Using Bodipy staining, we observed that *Brucella* infected cells displayed high lipid storage. A high lipid body count in infected cells is induced by several intracellular pathogens such as *M*. *tuberculosis* (reviewed in [50]). Lipid body are frequently found in close association with pathogen-containing phagosomes and lipid transfers have been observed between lipid body and pathogens such as *M*. *tuberculosis*, suggesting that bacterial pathogens induce the accumulation of lipids to use them as a source of energy and carbon. Interestingly, it has been recently shown in a model of *M*. *tuberculosis* [51] that lipid accumulating in macrophages can also favor bacterial latency through the reversible blockage of bacterial division. Little is known about the role of lipid metabolism during *Brucella* infection in mice. Cholesterol and ganglioside GM1, two components of lipid rafts, are implicated in the entry and short-term survival of *Brucella* in murine macrophages [52]. Mice deficient for the Niemann-Pick C1 (NCP1) gene controlling the trafficking of cholesterol-associated microdomains are resistant to *B*. *abortus* infection [53]. Our results suggest that long-term survival of *Brucella in vivo* may require high lipid content in host reservoir cells.

In summary, our results have further defined the phenotype of the main cell type favoring the persistence of *Brucella melitensis* in the spleen of IL-12p40^-/-^ susceptible mice. The preferential persistence of *Brucella* in Arg1^+^ lipid rich cells in our experimental model suggests that it could be interesting to more thoroughly analyze the impact of arginase activity, polyamines and lipid nutrients on the *Brucella* cell cycle. These data could help to develop new therapeutic strategies to control *Brucella* infection by specifically targeting natural reservoir cells.

## Supporting Information

S1 Fig Confirmation of IL12p40 and STAT6 deficiency in the group of BALB/c mice used for the experiments. The data are the PCR products of mice DNA on agarose gel. **A**, PCR amplification of STAT6 gene with the lanes from the left to the right being: ladder, DNA of wt, STAT6-deficient mice, IL12p40-deficient mice, STAT6/IL12p40-deficient mice, ladder. The amplification in wt and IL12p40-deficient mice is 275 base pairs (bps) long and the amplification in STAT6 and STAT6/IL12p40-deficient mice is 380 bps long. **B**, PCR amplification of IL12p40 gene with the lanes from the left to the right being: ladder, DNA of wt mice, STAT6-deficient mice, IL12p40-deficient mice, STAT6/IL12p40-deficient mice, ladder. The amplification in wt and STAT6-deficient mice is 687 base pairs (bps) long and the amplification in IL12p40 and STAT6/IL12p40-deficient mice is about 1600 bps long.
(TIF)

S2 Fig Characterization of the cell surface phenotype of infected cells in the spleen of IL12p40-deficient BALB/c mice. IL12p40-deficient BALB/c mice were injected i.n. with 2x10^7^ CFU of mCherry-Br. The mice were sacrificed at 28 days post-infection and the spleens were collected and examined by immunohistofluorescence. The left panels show the overall distribution of the F4/80-, GR1-, CD11b- and CD8α-expressing cells in the spleen. The panels to the right of the first ones show mCherry-Br co-localization with negative cells, weakly positive cells and highly positive cells for F4/80-, GR1, CD11b and CD8α. The panels are color-coded with the text for phalloidin, the antigen examined or mCherry-Br. Scale bar = 200 and 20 μm, as indicated. r.p.: red pulp; w.p.: white pulp. Yellow arrowheads indicate the presence of bacteria. The data are representative of at least three independent experiments.
(TIF)

S3 Fig Characterization of MHCII and CD11c expression on infected cells in the spleen of IL12p40-deficient BALB/c mice. IL12p40-deficient BALB/c mice were injected i.n. with 2x10^7^ CFU of mCherry-Br. The mice were sacrificed at 28 days post-infection and the spleens were collected and examined by immunohistofluorescence. The upper panels show the overall distribution of MHCII-expressing cells in the spleen (left), mCherry-Br co-localization with negative cells for MHCII (middle) and mCherry-Br co-localization with cells weakly expressing MHCII (right). The panels below show mCherry-Br co-localization with negative cells for MHCII (left) and co-localization of mCherry-Br and CD11c-expressing cells negative for MHCII (right). The panels are color-coded with the text for DAPI, the antigen examined or mCherry-Br. Scale bar = 200 and 20 μm, as indicated. r.p.: red pulp; w.p.: white pulp. Yellow arrowheads indicate the presence of bacteria. The data are representative of at least three independent experiments.
(TIF)

S4 Fig Co-localization between MOMA-1, CD11c and Arginase1 markers and mCherry-Br staining in the spleen of IL12p40-deficient BALB/c mice. IL12p40-deficient BALB/c mice were injected i.n. with 2x10^7^ CFU of mCherry-Br. The mice were sacrificed at 28 days post-infection and the spleens were collected and examined by immunohistofluorescence. **A**: The left panels show mCherry-Br co-localization with MOMA-1-expressing cells, the middle panels show mCherry-Br co-localization with CD11c-expressing cells and the right panel shows co-localization of MOMA-1- and CD11c-expressing cells. **B**: The left picture shows co-localization between Arg1-expressing cells and mCherry-Br, the right picture shows co-localization between Arg1-expressing cells and Bodipy staining. The panels are color-coded with the text for phalloidin, the antigen examined or mCherry-Br. Scale bar = 20 μm, as indicated. w.p.: white pulp. Yellow arrowheads indicate the presence of bacteria. The data are representative of at least two independent experiments.
(TIF)
