# Supplementary figures and images for: In Situ Characterization of Splenic Brucella melitensis Reservoir Cells during the Chronic Phase of Infection in Susceptible Mice
Source: PLoS One. 2015 Sep 16;10(9):e0137835. doi: 10.1371/journal.pone.0137835 (PMC4574346; doi:10.1371/journal.pone.0137835)

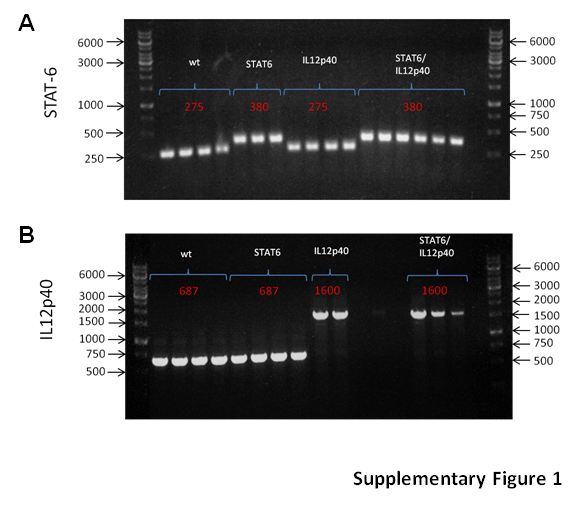

Supplement: S1 Fig — The data are the PCR products of mice DNA on agarose gel. A, PCR amplification of STAT6 gene with the lanes from the left to the right being: ladder, DNA of wt, STAT6-deficient mice, IL12p40-deficient mice, STAT6/IL12p40-deficient mice, ladder. The amplification in wt and IL12p40-deficient mice is 275 base pairs (bps) long and the amplification in STAT6 and STAT6/IL12p40-deficient mice is 380 bps long. B, PCR amplification of IL12p40 gene with the lanes from the left to the right being: ladder, DNA of wt mice, STAT6-deficient mice, IL12p40-deficient mice, STAT6/IL12p40-deficient mice, ladder. The amplification in wt and STAT6-deficient mice is 687 base pairs (bps) long and the amplification in IL12p40 and STAT6/IL12p40-deficient mice is about 1600 bps long. (TIF) [file pone.0137835.s001.tif]

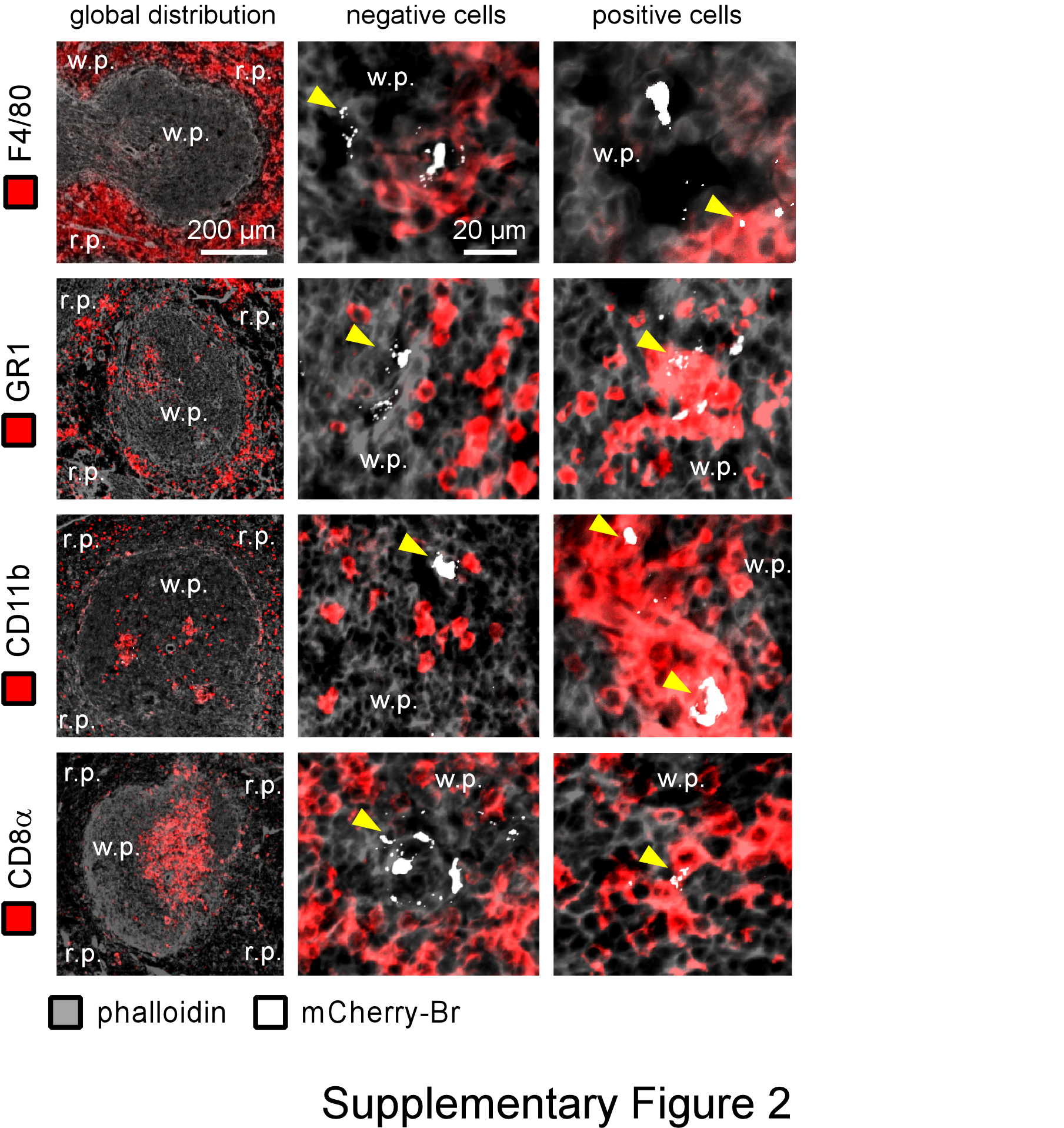

Supplement: S2 Fig — IL12p40-deficient BALB/c mice were injected i.n. with 2x10^7^ CFU of mCherry-Br. The mice were sacrificed at 28 days post-infection and the spleens were collected and examined by immunohistofluorescence. The left panels show the overall distribution of the F4/80-, GR1-, CD11b- and CD8α-expressing cells in the spleen. The panels to the right of the first ones show mCherry-Br co-localization with negative cells, weakly positive cells and highly positive cells for F4/80-, GR1, CD11b and CD8α. The panels are color-coded with the text for phalloidin, the antigen examined or mCherry-Br. Scale bar = 200 and 20 μm, as indicated. r.p.: red pulp; w.p.: white pulp. Yellow arrowheads indicate the presence of bacteria. The data are representative of at least three independent experiments. (TIF) [file pone.0137835.s002.tif]

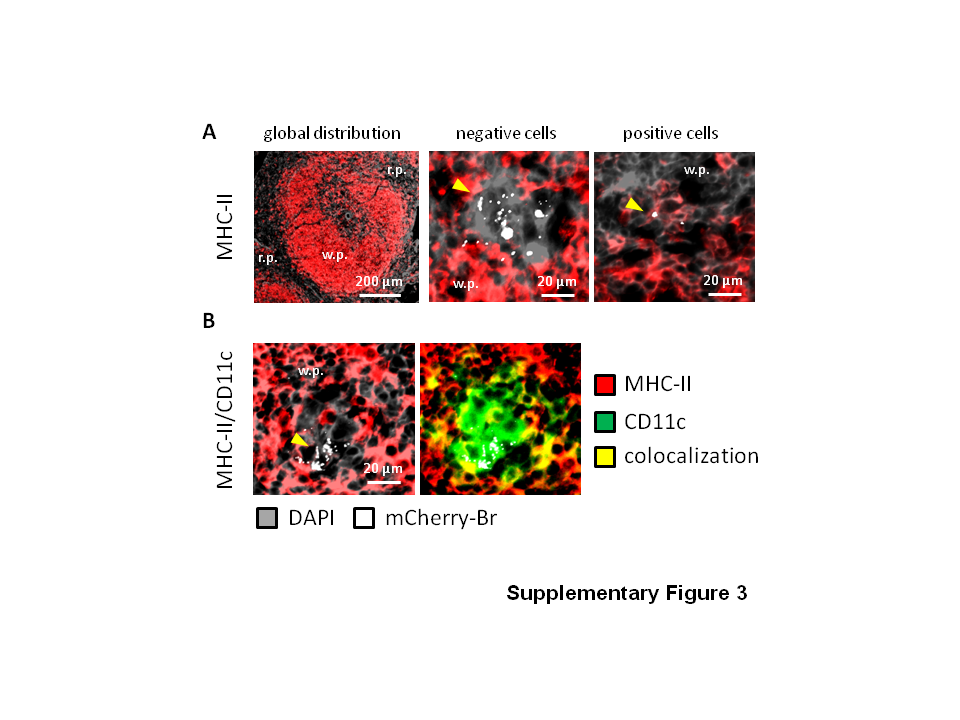

Supplement: S3 Fig — IL12p40-deficient BALB/c mice were injected i.n. with 2x10^7^ CFU of mCherry-Br. The mice were sacrificed at 28 days post-infection and the spleens were collected and examined by immunohistofluorescence. The upper panels show the overall distribution of MHCII-expressing cells in the spleen (left), mCherry-Br co-localization with negative cells for MHCII (middle) and mCherry-Br co-localization with cells weakly expressing MHCII (right). The panels below show mCherry-Br co-localization with negative cells for MHCII (left) and co-localization of mCherry-Br and CD11c-expressing cells negative for MHCII (right). The panels are color-coded with the text for DAPI, the antigen examined or mCherry-Br. Scale bar = 200 and 20 μm, as indicated. r.p.: red pulp; w.p.: white pulp. Yellow arrowheads indicate the presence of bacteria. The data are representative of at least three independent experiments. (TIF) [file pone.0137835.s003.tif]

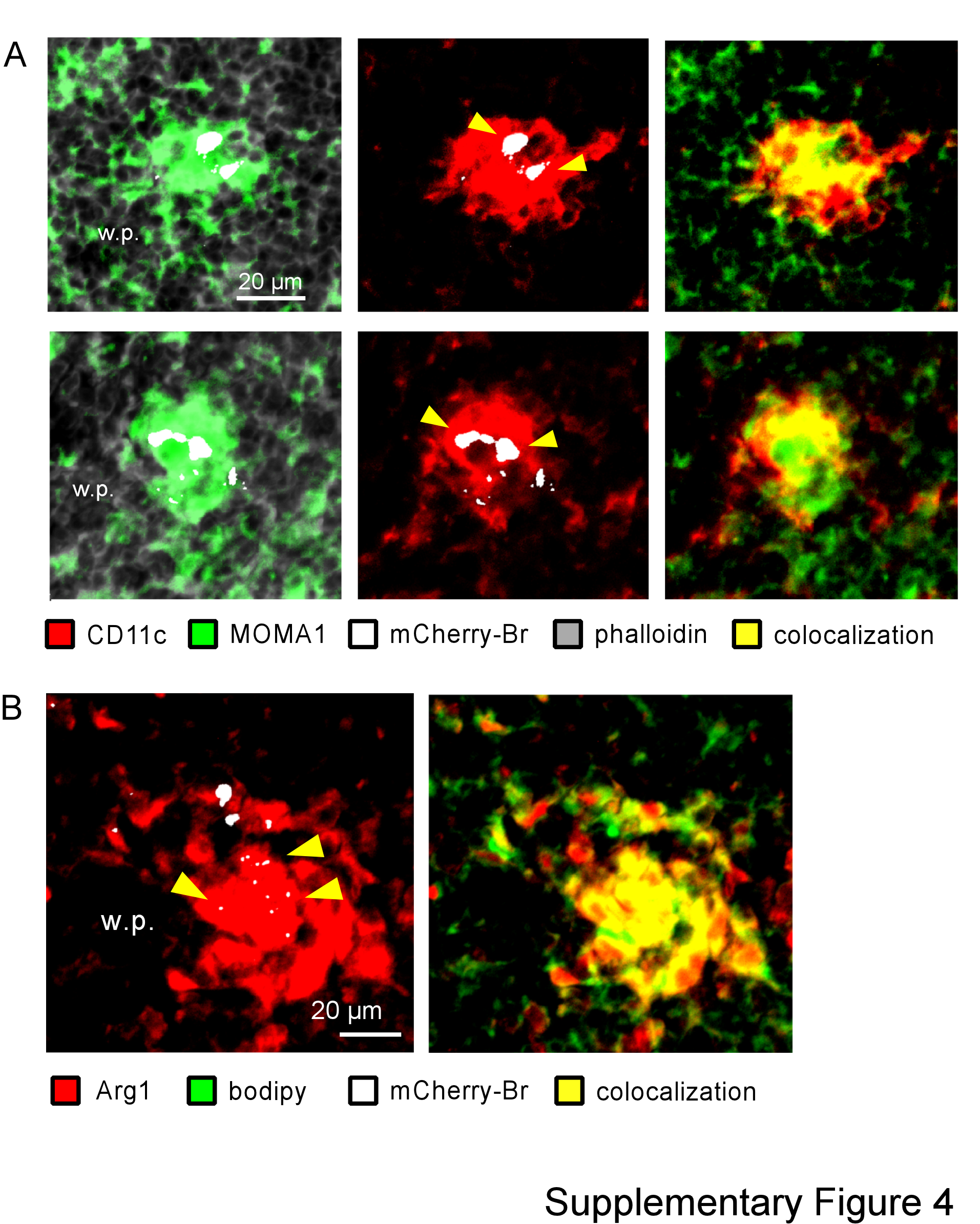

Supplement: S4 Fig — IL12p40-deficient BALB/c mice were injected i.n. with 2x10^7^ CFU of mCherry-Br. The mice were sacrificed at 28 days post-infection and the spleens were collected and examined by immunohistofluorescence. A: The left panels show mCherry-Br co-localization with MOMA-1-expressing cells, the middle panels show mCherry-Br co-localization with CD11c-expressing cells and the right panel shows co-localization of MOMA-1- and CD11c-expressing cells. B: The left picture shows co-localization between Arg1-expressing cells and mCherry-Br, the right picture shows co-localization between Arg1-expressing cells and Bodipy staining. The panels are color-coded with the text for phalloidin, the antigen examined or mCherry-Br. Scale bar = 20 μm, as indicated. w.p.: white pulp. Yellow arrowheads indicate the presence of bacteria. The data are representative of at least two independent experiments. (TIF) [file pone.0137835.s004.tif]
